# Association of Marital Status in the Testing and Treatment of Sexually Transmitted Infections in the Emergency Department

**DOI:** 10.7759/cureus.17489

**Published:** 2021-08-27

**Authors:** Hannah T Fox, Johnathan M Sheele

**Affiliations:** 1 Emergency Medicine, Mayo Clinic, Jacksonville, USA

**Keywords:** gonorrhea, chlamydia, trichomonas, married, maritial status, gender, emergency medicine, sexually transmitted infection, sexually transmitted disease, treatment

## Abstract

Introduction

Sexually transmitted infections (STIs) are frequently tested for and treated in the emergency department (ED). Age, race, and number of sexual partners are known risk factors for STIs. The objective of the current study was to examine marital status as it relates to testing and treating for STIs in the ED.

Methods

A database of 75,000 ED patient encounters from a single healthcare system in northeast Ohio between April 18, 2014, and March 7, 2017, was examined. All patients in the dataset underwent a urinalysis and urine culture or received STI testing in the ED. We performed Chi-square and multivariable regression analysis to examine the relationships between the patient's marital status and testing and treatment for STIs performed in the ED.

Results

There were 20,965 patient encounters where STI testing was performed and was analyzed. Patients were 9.1% (N=1,912) married, 86.6% (N=18,149) single, 4.0% (N=837) were neither married nor single, and 0.3% (N=67) with an unknown marital status. There were 7.1% (19/267) and 4.9% (12/267) of tested married men who were infected with gonorrhea and chlamydia, respectively, whereas only 0.4% (6/1,583) and 2.2% (35/1,588) of tested married women were infected with gonorrhea and chlamydia, respectively. Single men and women were both significantly more likely to have a positive test for gonorrhea and chlamydia compared to married men and women, respectively (P<0.001). Married men and women, compared to single men and women, respectively, were more likely to be given antibiotics for gonorrhea and chlamydia in the ED when the infection was present and not be given antibiotics for the infections when testing was negative (P<0.001). Single women (9.1%; 1,291/14,258) were more likely than married women (4.9%; 75/1,534) to have a positive test for trichomonas, but there were no significant differences between married (1.0%; 1/100) and single men (0.7%; 6/893).

Conclusion

Even when accounting for age and race, marital status can help predict infection with gonorrhea and chlamydia in the ED. The marital status could be considered by clinicians when risk stratifying patients regarding testing and treating for the diseases in the ED. Gonorrhea and chlamydia are much more common in single men and women and much less common in married persons. However, married men tested for gonorrhea and chlamydia were more than twice as likely to test positive for infection than married women. Married men and women were both more likely to be appropriately treated with antibiotics for gonorrhea and chlamydia in the ED (i.e., testing negative for infection and not receiving antibiotics or testing positive and receiving antibiotics) compared to non-married men and women. While trichomonas was more common in single women than married women, the infection was less common in men, and both married men and single men had similar rates of testing positive for the infection.

## Introduction

Sexually transmitted infections (STIs) such as gonorrhea and chlamydia are increasing in prevalence and recently have reached an all-time high in the United States (U.S.) [1]. This increase is happening concurrently with growing antibiotic resistance seen with gonorrhea [1]. Clinicians may need to provide empiric treatment for STIs in the emergency department (ED) because definitive STI testing results may not be available during the clinical encounter. Both overtreatment and undertreatment of STIs in the ED are common, and more accurate risk stratification for STIs in the ED could lead to better antibiotic stewardship [2-5].

Adolescents and young adults are at higher risk for STIs, but STIs occur in the "middle age" category of age 45-64 years [6,7]. There is an increasing percentage of both single and divorced adults and a decreasing percentage of married adults in the U.S. [8,9]. The changing marriage demographics in the U.S. could affect sexual behavior and the risk factors for acquiring an STI.

In many cultures, being married often culturally confers the presumption of monogamy and thus minimal risk of sexually transmitted disease [10]. Married people report fewer sexual partners and are less likely to engage in risky sexual behaviors than their unmarried counterparts [11-14]. However, in reality, monogamy is not always present or presumed among married partners [10]. In a recent study, the percent of married and cohabitating individuals in presumed monogamous relationships that reported sexual contact outside of their primary partnership ranged from 13.3% to 37.5%. The majority of these reported that their partner was unaware of their infidelity, and this group also had low condom use [15]. The prevalence of STIs among ED patients related to marital status is understudied. This study's objective was to examine differences in the rates and types of STIs in patients undergoing testing in the ED as it relates to marital status.

## Materials and methods

The study received institutional review board approval from University Hospitals (UH). A UH information technology (IT) team created a database of 75,000 consecutive UH ED patient encounters. Data were extracted from the UH electronic medical record (EMR) by IT, who created a custom structured query language (SQL) in SQL Server Management Studio (SSMS). UH IT was blinded to the specific study objectives. The database included patients ≥18 years of age who received testing for gonorrhea, chlamydia, or trichomonas or received a urinalysis and urine culture in the ED. All patient encounters took place between April 18, 2014, and March 7, 2017. For our study, only those who underwent nucleic acid amplification test (NAAT) for gonorrhea, chlamydia, or trichomonas, or who had a vaginal wet preparation for trichomonas were included in this analysis. Manuscripts have already been published from this dataset [16-22].

The documented marital status associated with the ED clinical encounter was used in the analysis. Patients were infected with *Chlamydia trachomatis* and *Neisseria gonorrhoeae* if they had a positive NAAT (APTIMA, Hologic). Patients were infected with *Trichomonas vaginalis* if the organism was seen in the urinalysis, on vaginal wet preparation, or had a positive NAAT. Patients were considered uninfected with *T. vaginalis* only if they had a negative NAAT. 

The mean number of urine white blood cells (WBCs) were used in the analysis if the clinical laboratory provided a range, and WBCs >100 were changed to 101 cells/high-powered field (HPF) for the analysis. Vaginal wet preparation WBCs were reported as 0-5, 5-10, 11-15, 15-25, 25-50, and 50-100 cells/HPF from the clinical laboratory, and ≤10 and >10 WBC/HPF was used in the analysis. Patients given ceftriaxone or cefixime plus azithromycin or who received a discharge prescription for doxycycline were considered correctly treated for gonorrhea and chlamydia. Patients were considered inappropriately treated for gonorrhea and chlamydia in the ED if they tested negative for the disease but were treated with antibiotics for the infection or they were not treated for gonorrhea and chlamydia but tested positive for the infection. Missing and erroneous variables were not included in the analysis.

Data analysis

Categorical variables were summarized as counts and percentages and analyzed using the Chi-square test. Continuous variables were summarized as the median and IQR and were analyzed using the Wilcoxon rank sum test. Multivariable logistic regression analyses accounted for age (years) and Black race (vs. non-Black race). For analyses examining if women were correctly treated for gonorrhea and chlamydia, the following additional variables were included in the analysis: urine leukocyte esterase (0-3+), urine WBCs, and the vaginal wet preparation: WBCs, yeast, and clue cells. Odds ratios were calculated, and all statistical tests were two-sided. P-values <0.05 were considered statistically significant. Analyses were conducted using JMP Pro 14.

## Results

There were 20,965 patient encounters included in the analysis. Patients were: married 9.12% (N=1,912), had a life partner 0.05% (N=10), single 86.57% (N=18,149), widowed 0.46% (N=97), divorced 2.62% (N=550), separated 0.86% (N=180), and with an unknown marital status 0.32% (N=67). There were 85.06% (N=17,832) of patients who were female, the median age of was 26 (22.33), and 88.08% (N=18,380) were Black/African American. The overall rates of infection were: 4.52% (928/20,509) for gonorrhea; 8.86% (1,817/20,511) for chlamydia; and 26.55% (1,976/7,442) for trichomonas. Demographic and patient characteristics for men and women related to their marital status are summarized in Tables 1-3.

**Table 1:** Demographic and Patient Characteristics of Women in the Analysis ED: emergency department; HPF: high-powered field; N: number; NAAT: nucleic acid amplification test; WBC: white blood cell; %: percentage. Patients with unknown marital statuses were not included.

	Married 9.3% (N=1645)	Life partner 0.05% (N=9)	Single 86.6% (N=15402)	Widowed 0.5% (N=93)	Divorced 2.7% (N=482)	Separated 0.8% (N=150)
Age, years (N=17,781)	33 (28.40)	32 (28.40)	25 (22.31)	59 (43.74)	41 (34.48)	35 (30.44)
Black race/ethnicity, % (N=17,718)	1109/1636 (67.79)	7/9 (77.78)	13,995 (91.16)	77/93 (82.80)	336/478 (70.29)	121/150 (80.67)
Urine						
WBCs, cells/HPF (N=11452)	3 (3.13)	15 (5.25)	5 (3.15)	12 (3.58)	4 (3.13)	4 (3.13)
Leukocyte esterase (N=15585)	0 (0.1)	0 (0.2)	0 (0.1)	1 (0.2)	0 (0.1)	0 (0.1)
Trichomonas, % (N=11376)	21/1,1016 (2.07)	0/4 (0)	265/9,894 (2.68)	5/62 (8.06)	2/305 (0.66)	1/95 (1.05)
Vaginal wet preparation						
>10 WBC/HPF, % (N=17066)	451/1,579 (28.56)	5/8 (62.50)	4,967/14,780 (33.61)	26/91 (28.57)	123/462 (26.62)	48/146 (32.88)
Clue cells, % (cells/HPF) (N=16685)	498/1,541 (32.32)	2/8 (25.00)	6,442/14,449 (44.58)	31/89 (34.83)	186/458 (40.61)	51/140 (36.43)
Yeast, % (N=16484)	107/1,535 (6.97)	2/8 (25.00)	951/14,254 (6.67)	2/89 (2.25)	24/457 (5.25)	16/141 (11.35)
*T. vaginalis*, % (N=16488)	75/1,534 (4.89)	1/8 (12.50)	1,291/14,258 (9.05)	6/90 (6.67)	29/457 (6.35)	19/141 (13.48)
*N. gonorrhoeae* NAAT, % (N=17337)	6/1,583 (0.38)	0/9 (0)	495/15,059 (3.29)	1/82 (1.22)	5/459 (1.09)	2/145 (1.38)
*C. trachomatis* NAAT, % (N=17381)	35/1,588 (2.20)	0/9 (0)	1,304/15,047 (8.67)	0/82 (0)	15/459 (3.27)	5/145 (3.45)
*T. vaginalis* NAAT, % (N=5180)	95/502 (18.92)	1/6 (16.67)	1,741/5,865 (29.68)	7/28 (25.00)	39/173 (22.54)	23/61 (37.70)
Correctly treated for gonorrhea and chlamydia in the ED (infected patients received appropriate antibiotics and uninfected patients received no treatment for those infections (N=17,360)	1397 (87.97)	7 (77.78)	11,518 (76.40)	79 (96.34)	360 (78.26)	111 (76.55)

**Table 2:** Demographic and Patient Characteristics of Men in the Analysis ED: emergency department; HPF: high-powered field; N: number; NAAT: nucleic acid amplification test; WBC: white blood cell; %: percentage. Patients with unknown marital statuses were not included.

	Married 8.5% (N=267)	Life partner	Single 87.7% (N=2747)	Widowed 0.1% (N=4)	Divorced 2.2% (N=68)	Separated 1% (N=30)
Age, years (N=3117)	39 (32.48)	48 (48.48)	26 (22.34)	67 (50.84)	50 (42.57)	38 (32.43)
Black race/ethnicity, % (N=3113)	189/264 (71.59)	1/1 (100)	2,448/2,736 (89.47)	2/4 (50.00%)	46/68 (67.65)	19/30 (63.33)
Urine						
WBCs, cells/HPF (N=1045)	9 (3.36)	NA	13 (3.62)	45 (12.94)	12.5 (3.36)	3 (2.13)
Leukocyte esterase (N=1841)	0 (0.1)	0 (0.0)	0 (0.1)	3 (1.3)	0 (0.1)	0 (0.1)
Trichomonas (N=1036)	1/100 (1.00%)	0/0 (0)	6/893 (0.67)	0/4 (0)	0/29 (0)	0/10 (0)
*N. gonorrhoeae* NAAT, % (N=3105)	19/267 (7.12)	0/1 (0)	396/2,736 (14.47)	0/4 (0)	1/67 (1.49)	2/30 (6.67)
*C. trachomatis* NAAT, % (N=3114)	13/267 (4.87)	0/1 (0)	438/2,744 (15.96)	0/4 (0)	2/68 (2.94)	1/30 (3.33)
*T. vaginalis* NAAT, % (N=778)	5/42 (11.90)	0/1 (0)	55/718 (7.66)	0/0 (0)	1/13 (7.69)	0/4 (0)
Infected with *T. vaginalis*, % (N=785)	6/43 (13.95)	0/1 (0)	61/724 (8.43)	0/0 (0)	1/13 (7.69)	0/4 (0)
Correctly treated for gonorrhea and chlamydia in the ED (infected patients received appropriate antibiotics and uninfected patients received no treatment for those infections; N=3,116)	163 (61.05)	1 (100)	1,478 (53.82)	3 (75.00)	44 (64.71)	18 (60.00)

**Table 3:** Rates of Gonorrhea, Chlamydia, and Trichomonas and Marital Status ^Treated for gonorrhea and chlamydia in the ED and were infected with either or both bacteria or was not given antibiotics for gonorrhea and chlamydia in the ED and they were not infected with either bacteria. ED: emergency department; NAAT: nucleic acid amplification test. Only considered negative for gonorrhea, chlamydia, and trichomonas if they had a corresponding negative NAAT.

	Gonorrhea	Chlamydia	Trichomonas	Correctly treated for gonorrhea and chlamydia in the ED^
Women	510/17,388 (2.93)	1,360/17,381 (7.82)	1,908/6,652 (28.68)	13,517/17,411 (77.63)
Single	495/15,059 (3.29)	1,304/15,047 (8.67)	1,741/5,865 (29.68)	11,518/15,076 (76.40)
Married	6/1,583 (0.38)	35/1,588 (2.20)	95/502 (18.92)	1,397/1,588 (87.97)
Divorced	5/459 (1.09)	15/459 (3.27)	39/173 (22.54)	360/460 (78.26)
Widowed	1/82 (1.22)	0/82 (0)	7/28 (25.00)	79/82 (96.34)
Separated	2/145 (1.38)	5/145 (3.45)	23/61 (37.70)	111/145 (76.55)
Life partner	0/9 (0)	0/9 (0)	1/6 (16.67)	7/9 (77.78)
Unknown	1/51 (1.96)	1/51 (1.96)	2/17 (11.76)	45/51 (88.24)
Men	418/3,121 (13.39)	457/3,130 (14.60)	68/790 (8.61)	1,714/3,132 (54.73)
Single	396/2,736 (14.47)	438/2,744 (15.96)	61/724 (8.43)	1,478/2,746 (53.82)
Married	19/267 (7.12)	13/267 (4.87)	6/43 (13.95)	163/267 (61.05)
Divorced	1/67 (1.49)	2/68 (2.94)	1/13 (7.69)	44/68 (64.71)
Widowed	0/4 (0)	0/4 (0)	0/0 (0)	3/4 (75.00)
Separated	2/30 (6.67)	1/30 (3.33)	0/4 (0)	18/30 (60.00)
Life partner	0/1 (0)	0/1 (0)	0/1 (0)	1/1 (100)
Unknown	0/16 (0)	3/16 (18.75)	0/5 (0)	7/16 (43.75)

Women and gonorrhea

There were 2.93% (510/17,388) women infected with gonorrhea of which 97.06% were single, 0.98% divorced, 0.20% widowed, 0.39% separated, 1.18% married, 0% with a life partner, and 0.20% had an unknown marital status (P<0.001; Table 4).

**Table 4:** Marital Status and Gonorrhea Adjusted analysis was adjusted for age and race. CI: confidence interval; ED: emergency department; OR: odds ratio; vs: versus; %: percentage.

	OR (95% CI)	P-value	Adjusted OR (95% CI)	Adjusted P-value
Women				
Single vs married	8.93 (3.99–20.01)	<0.001	4.21 (1.87-9.52)	<0.001
Single vs divorced	3.09 (1.27–7.48)	0.005	0.91 (0.37–2.25)	0.83
Single vs widowed	2.75 (0.38–19.82)	0.53	0.36 (0.05–2.69)	0.32
Single vs separated	2.43 (0.60–9.84)	0.34	1.02 (0.25–4.19)	0.97
Married vs divorced	0.35 (0.10–1.14)	0.08	0.21 (0.06–0.73)	0.01
Married vs widowed	0.31 (0.04–2.59)	0.30	0.07 (0.007–0.72)	0.03
Married vs separated	0.27 (0.05–1.36)	0.14	0.23 (0.04–1.16)	0.08
Divorced vs widowed	0.89 (0.10–7.74)	>0.99	0.32 (0.03–3.11)	0.33
Divorced vs separated	0.79 (0.15–4.10)	0.68	1.06 (0.20–5.69)	0.95
Widowed vs separated	0.88 (0.08–9.89)	>0.99	15.82 (0.56–450.48)	0.11
Single vs non-single	5.50 (3.23–9.37)	<0.001	2.34 (1.35–4.04)	0.002
Married vs non-married	0.12 (0.05–0.26)	<0.001	0.23 (0.10–0.53)	<0.001
Divorced vs non-divorced	0.36 (0.15–0.87)	0.02	1.24 (0.50–3.06)	0.64
Widowed vs non-widowed	0.41 (0.06–2.93)	0.74	3.21 (0.43–24.24)	0.26
Separated vs non-separated	0.46 (0.11–1.86)	0.45	1.08 (0.26–4.41)	0.92
Men				
Single vs married	2.21 (1.37–3.56)	<0.001	1.09 (0.66–1.81)	0.73
Single vs non-single	2.67 (1.71–4.16)	<0.001	1.19 (0.74–1.92)	0.47
Married vs non-married	0.47 (0.29–0.76)	0.001	0.95 (0.57–1.57)	0.83
Divorced vs non-divorced	0.10 (0.01–0.69)	0.002	0.31 (0.04–2.30)	0.25
Separated vs non-separated	0.46 (0.11–1.92)	0.42	0.89 (0.21–3.81)	0.87

On univariable analysis, single compared to married, single compared to non-single, and single compared to divorced had significantly higher odds of testing positive for infection with gonorrhea (P≤0.005). Married compared to non-married and divorced compared to non-divorced had significantly lower odds of testing positive for infection with gonorrhea (P≤0.02 for both). On multivariable analysis, single compared to married and single compared to non-single had significantly higher odds of testing positive for infection with gonorrhea (P≤0.002 for both). Married compared to divorced, married compared to widowed, and married compared to non-married were significantly less likely to be diagnosed with gonorrhea (P≤0.03 for all).

Women and chlamydia

There were 7.82% (1,360/17,381) of women infected with chlamydia, of which 95.88% were single, 1.10% were divorced, 0% were widowed, 0.37% were separated, 2.57% were married, 0% had a life partner, and 0.07% had an unknown marital status (P<0.001). On univariable analysis, single compared to married, single compared to divorced, single compared to separated, and single compared to non-single were significantly more likely to be infected with chlamydia (P≤0.02 for all; Table 5).

**Table 5:** Marital Status and Chlamydia Adjusted analysis was adjusted for age and race. CI: confidence interval; ED: emergency department; OR: odds ratio; vs: versus; %: percentage.

	OR (95% CI)	P-value	Adjusted OR (95% CI)	Adjusted P-value
Women				
Single vs married	4.21 (3.00–5.91)	<0.001	1.88 (1.33–2.67)	<0.001
Single vs divorced	2.81 (1.67–4.71)	<0.001	0.74 (0.42–1.29)	0.28
Single vs separated	2.66 (1.09–6.49)	0.02	0.95 (0.38–2.36)	0.91
Married vs divorced	0.67 (0.36–1.23)	0.23	0.47 (0.24–0.90)	0.02
Married vs separated	0.63 (0.24–1.64)	0.38	0.55 (0.21–1.44)	0.22
Divorced vs separated	0.95 (0.34–2.65)	>0.99	1.17 (0.40–3.38)	0.77
Single vs non-single	3.84 (2.92–5.05)	<0.001	1.55 (1.16–2.07)	0.003
Married vs non-married	0.25 (0.17–0.34)	<0.001	0.52 (0.37–0.74)	<0.001
Divorced vs non-divorced	0.39 (0.23–0.65)	<0.001	1.44 (0.83–2.52)	0.20
Separated vs non-separated	0.42 (0.17–1.02)	0.04	1.08 (0.43–2.68)	0.87
Men				
Single vs married	3.71 (2.11–6.54)	<0.001	1.51 (0.83–2.73)	0.18
Single vs non-single	4.20 (2.52–7.01)	<0.001	1.52 (0.88–2.62)	0.13
Married vs non-married	0.28 (0.16–0.49)	<0.001	0.68 (0.37–1.23)	0.20
Divorced vs non-divorced	0.17 (0.04–0.71)	0.003	0.92 (0.22–3.94)	0.91
Separated vs non-separated	0.20 (0.03–1.47)	0.11	0.44 (0.06–3.28)	0.42

Married compared to non-married, divorced compared to non-divorced, and separated compared to non-separated were significantly less likely to test positive for chlamydia (P≤0.04 for all). On multivariable analysis, single compared to married and single compared to non-single women were significantly more likely to test positive for chlamydia (P≤0.003). Married compared to divorced, and married compared to non-married, were significantly less likely to test positive for chlamydia (P≤0.02).

Women and trichomonas

There were 28.68% (1,908/6,652) of women with known marital status infected with *T. vaginalis*, of which 91.25% were single, 2.04% were divorced, 0.37% were widowed, 1.21% were separated, 4.98% were married, 0.05% had a life partner, and 0.10% had an unknown marital status (P<0.001). On univariable analysis, single compared to married, single compared divorced, and single compared to non-single women were significantly more likely to test positive for *T. vaginalis* (P≤0.04 for all; Table 6).

**Table 6:** Marital Status and Trichomonas Adjusted analysis was adjusted for age and race. CI: confidence interval; ED: emergency department; OR: odds ratio; vs: versus; %: percentage.

	OR (95% CI)	P-value	Adjusted OR (95% CI)	Adjusted P-value
Women				
Single vs married	1.81 (1.44–2.28)	<0.001	1.88 (1.48–2.40)	<0.001
Single vs divorced	1.45 (1.01–2.08)	0.04	1.81 (1.23–2.66)	0.003
Single vs widowed	1.27 (0.54–2.98)	0.68	2.73 (1.11–6.71)	0.03
Single vs separated	0.70 (0.41–1.17)	0.20	0.92 (0.54–1.58)	0.76
Married vs divorced	0.80 (0.53–1.22)	0.32	0.91 (0.58–1.42)	0.67
Married vs widowed	0.70 (0.29–1.70)	0.46	1.33 (0.48–3.69)	0.58
Married vs separated	0.39 (0.22–0.68)	0.001	0.51 (0.28–0.91)	0.02
Divorced vs widowed	0.87 (0.35–2.21)	0.81	0.73 (0.26–2.04)	0.54
Divorced vs separated	0.48 (0.26–0.90)	0.03	0.49 (0.26–0.94)	0.03
Widowed vs separated	0.55 (0.20–1.50)	0.33	0.54 (0.17–1.69)	0.29
Single vs non-single	1.55 (1.29–1.86)	<0.001	1.74 (1.42–2.12)	<0.001
Married vs non-married	0.56 (0.44–0.70)	<0.001	0.56 (0.44–0.72)	<0.001
Divorced vs non-divorced	0.72 (0.50–1.03)	0.07	0.63 (0.43–0.93)	0.02
Widowed vs non-widowed	0.83 (0.35–1.95)	0.83	0.47 (0.19–1.14)	0.09
Separated vs non-separated	1.51 (0.90–2.54)	0.12	1.28 (0.75–2.18)	0.36
Men				
Single vs married	0.57 (0.23–1.40)	0.26	0.74 (0.31–2.07)	0.53
Single vs non-single	0.71 (0.31–1.63)	0.47	1.03 (0.42–2.48)	0.95
Married vs non-married	1.78 (0.72–4.38)	0.26	1.43 (0.56–3.63)	0.45
Divorced vs non-divorced	0.88 (0.11–6.85)	>0.99	0.47 (0.06–3.81)	0.48

Married compared to separated, divorced compared to separated, and married compared to non-married were significantly less likely to test positive for *T. vaginalis* (P≤0.03 for all). On multivariable analysis, single compared to married, single compared to divorced, single compared to widowed, and single compared to non-single were significantly more likely to test positive for *T. vaginalis* (P≤0.03 for all). Married compared to separated, divorced compared to separated, married compared to non-married, and divorced vs. non-divorced all had lower odds of testing positive for* T. vaginalis* (P≤0.03 for all).

Women correctly treated for gonorrhea and chlamydia in the ED

On univariable and multivariable analyses, married compared to divorced, married compared to separated, widowed compared to separated, married compared to non-married, and widowed compared to non-widowed were significantly more likely to be correctly treated for gonorrhea and chlamydia in the ED (P≤0.048 for all), with the exception being widowed compared to separated on multivariable analysis (P=0.06; Table 7).

**Table 7:** Marital Status and Correctly Treated for Gonorrhea and Chlamydia in the ED Adjusted analysis adjusted for age and race for men and for women: age, race, urine WBCs, urine leukocyte esterase, and from the vaginal wet preparation: WBCs, clue cells, yeast, and trichomonas. Infected patients received appropriate antibiotics and uninfected patients received no treatment for those infections. CI: confidence interval; ED: emergency department; OR: odds ratio; WBCs: white blood cells; vs: versus; %: percentage.

	OR (95% CI)	P-value	Adjusted OR (95% CI)	Adjusted P-value
Women				
Single vs married	0.44 (0.38–0.52)	<0.001	0.61 (0.49–0.75)	<0.001
Single vs divorced	0.90 (0.72–1.13)	0.37	1.19 (0.87–1.63)	0.28
Single vs widowed	0.12 (0.04–0.39)	<0.001	0.22 (0.05–0.94)	0.04
Single vs separated	0.99 (0.67–1.46)	>0.99	1.44 (0.87–2.38)	0.16
Married vs divorced	2.03 (1.55–2.66)	<0.001	1.85 (1.26–2.70)	0.002
Married vs widowed	0.28 (0.09–0.89)	0.02	0.23 (0.05–1.07)	0.06
Married vs separated	2.24 (1.48–3.39)	<0.001	2.13 (1.21–3.73)	0.008
Divorced vs widowed	0.14 (0.04–0.44)	<0.001	0.23 (0.05–1.06)	0.06
Divorced vs separated	1.10 (0.71–1.72)	0.65	1.15 (0.63–2.12)	0.65
Widowed vs separated	8.07 (2.39–27.19)	<0.001	5.89 (0.96–36.08)	0.06
Single vs non-single	0.55 (0.48–0.62)	<0.001	0.75 (0.63–0.89)	0.001
Married vs non-married	2.24 (1.92–2.62)	<0.001	1.65 (1.34–2.03)	<0.001
Divorced vs non-divorced	1.04 (0.83–1.30)	0.78	0.76 (0.56–1.04)	0.09
Widowed vs non-widowed	7.64 (2.41–24.21)	<0.001	4.24 (1.01–17.80)	0.048
Separated vs non-separated	0.94 (0.64–1.39)	0.76	0.65 (0.40–1.08)	0.10
Men				
Single vs married	0.74 (0.58–0.96)	0.02	0.69 (0.53–0.92)	0.01
Single vs non-single	0.72 (0.57–0.90)	0.004	0.67 (0.53–0.86)	0.002
Married vs non-married	1.32 (1.02–1.71)	0.03	1.35 (1.03–1.78)	0.03
Divorced vs non-divorced	1.53 (0.92–2.52)	0.11	1.61 (0.95–2.71)	0.08
Widowed vs non-widowed	2.48 (0.26–23.86)	0.63	2.59 (0.26–25.71)	0.42
Separated vs non-separated	1.24 (0.60–2.58)	0.59	1.16 (0.55–2.43)	0.70

Single compared to married, single compared to widowed, divorced compared widowed, and single compared to non-single were all significantly less likely to be correctly treated for gonorrhea and chlamydia in the ED (P≤0.02 for all). On univariable and multivariable regression analyses, single compared to married, single compared to widowed, married compared to widowed, divorced compared to widowed, single compared to non-single were significantly less likely to be correctly treated in the ED for gonorrhea and chlamydia (P≤0.04 for all) except for married compared to widowed and divorced compared to widowed on multivariable analysis (P=0.06 for both).

Men and gonorrhea

There were 13.46% (418/3105) men with a known marital status infected with gonorrhea, of which 94.74% were married, 0.24% were divorced, 0% were widowed, 0.48% were separated, 4.55% were married, and 0% had a life partner (Table 2). On univariable analysis, single compared to married and single compared to non-single were significantly more likely to test positive for gonorrhea (P≤0.001 for both; Table 4). Married compared to non-married and divorced compared non-divorced were significantly less likely to test positive for gonorrhea (P≤0.002 for both). No associations were significant on multivariable analysis.

Men and chlamydia

There were 14.57% (454/3,114) men with known marital status infected with chlamydia, of which 96.84% were single, 0.44% were divorced, 0% were widowed, 0.22% were separated, 2.84% were married, and 0% had a life partner. On univariable analysis, single compared to married and single compared to non-single were significantly more likely to test positive for chlamydia (P≤0.001 for both; Table 5). Married compared to non-married and divorced compared to non-divorced were significantly less likely to test positive for chlamydia (P≤0.003 for both). No associations were significant on multivariable analysis.

Men and trichomonas

There were 9.48% (68/717) men with known marital status infected with trichomonas, of which 89.71% were single, 1.47% divorced, 0% widowed, 0% separated, 8.82% married, and 0% with a life partner. No univariable or multivariable associations were significantly associated with a positive *T. vaginalis* test (Table 6).

Men correctly treated for gonorrhea and chlamydia in the ED

On both univariable and multivariable analysis, single compared to married and single compared to non-single were significantly less likely to be correctly treated for gonorrhea and chlamydia (P≤0.02 for all; Table 7). On both univariable and multivariable analysis, married compared to non-married was significantly associated with being correctly treated for gonorrhea and chlamydia in the ED (P=0.03 for both).

Both genders and marital status

On multivariable analysis, married and single women were significantly less likely to have a positive test for gonorrhea and chlamydia than married and single men, respectively (P≤0.001 for both; Table 8).

**Table 8:** Multivariable Regression Analysis Examining Gender and Marital Status Adjusted analysis adjusted for age and race. CI: confidence interval; ED: emergency department; OR: odds ratio; %: percentage.

	Adjusted OR (95% CI); P-value for married women vs married men	Adjusted OR (95% CI); P-value for single women vs single men
+ Gonorrhea	0.04 (0.02–0.11); <0.001>	0.18 (0.15–0.20); <0.001
+ Chlamydia	0.32 (0.17–0.63); <0.001	0.43 (0.38–0.48); <0.001
+ Trichomonas	1.91 (0.77–4.72); 0.16	4.99 (3.80–6.55); <0.001
Correctly treated for gonorrhea and chlamydia in the ED (infected patients received appropriate antibiotics and uninfected patients received no treatment for those infections	4.75 (3.52–6.42); <0.001	2.86 (2.62–3.11); <0.001

Married and single women were significantly more likely to be correctly treated for gonorrhea and chlamydia in the ED than married and single men, respectively (P≤0.001 for both. Single women were significantly more likely to have a positive test for trichomonas than single men (P≤0.001 for all). There were no significant differences in the odds of married women and married men and having a positive test for trichomonas (P=0.16).

## Discussion

Both gonorrhea and chlamydia were more prevalent among single men and women and were less prevalent in divorced, widowed, separated, and married ED patients. Married men and women were also significantly less likely to be inappropriately treated for an STI, even when accounting for the potential confounders of age and race.

More women in the dataset were screened for STIs than men, which could be due to more women presenting to the ED with genitourinary complaints. Women are much more likely to contract an STI during heterosexual intercourse, and women have higher rates of STIs than men [23]. Interestingly, the rates of gonorrhea and chlamydia among married men were more than double those seen in married women. It is possible that married men, compared to married women, were less likely to have an established primary care physician who could screen for and treat symptomatic STIs. Additionally, men report higher rates of nonmonogamy than women and are more likely to have symptomatic infections [24,25]. Clinician bias could also affect our results because physicians may be uncomfortable discussing HIV and STI prevention with older women, and older women may be uncomfortable initiating these conversations with healthcare providers [26,27].

Limits

Our data represented a single health system in northeast Ohio, and there may have been some socioeconomic homogeneity within the dataset. Some ED patients, especially men, may have been empirically treated with antibiotics for STIs without testing, and they would not have been included in our dataset. Not all women undergoing a vaginal wet prep or testing for gonorrhea and chlamydia had a *T. vaginalis* NAAT performed, suggesting selection bias by the clinician. Divorced, widowed, and separated patients were infrequently tested for STIs in our dataset. Our study population included a significantly higher percentage of females than male patients, which has also been observed in other STI studies from the ED [3,28]. ED providers may have been aware of the patient's marital status in the ED, which could have biased their workup and treatment.

## Conclusions

Having a positive test for gonorrhea and chlamydia in the ED is associated with marital status. Both single male and female ED patients are more likely to test positive for gonorrhea and chlamydia than with similar genders of a different marital categorization. Married men were more than twice as likely as married women to have a positive test for gonorrhea and chlamydia in the ED.
